# Mortality Rates Among U.S. Service Members, 2010–2020

**Published:** 2026-04-01

**Authors:** Shauna L. Stahlman, Brianna L. Rupp, Chiping Nieh, John C. Walsh, Parrish P. Balcena, Natalie Y. Wells

**Affiliations:** Epidemiology and Analysis Branch, Armed Forces Health Surveillance Division, Public Health Directorate, Defense Health Agency, Silver Spring, MD: Dr. Stahlman, Dr. Nieh, Dr. Wells; Armed Forces Medical Examiner System, Dover, DE: Dr. Rupp, Dr. Balcena; Naval Construction Group TWO, Gulfport, MS: Dr. Walsh

## Abstract

This report updates previous summaries of the numbers, rates, trends and causes of death among U.S. active component, National Guard, and reserve component members from 2010 through 2020. Mortality rates among service members in all components decreased from 2011 to 2014, corresponding with a drawdown of U.S. military operations in U.S. Central Command. Compared to their respective counterparts, all-cause mortality rates were highest in the guard and reserve component and among Army soldiers, male service members, non-Hispanic White individuals, those in the oldest age category (age 55 years and older), and service members in combat-related occupations. Suicide and self-inflicted injury was the leading cause of death for both U.S. service women and men. Mortality rates for all causes of death among military service members were lower than the in U.S. population after adjustments for age, sex, and race and ethnicity—with the exception of suicide and self-inflicted injury, for which rates were higher. These findings demonstrate the need for a continued emphasis on suicide prevention programs to improve service member well-being. By identifying the specific subpopulations at highest risk for various causes of mortality, these surveillance data provide information for the Department of War to refine and more effectively target its prevention efforts and resources. Continued mortality surveillance is essential to identify emerging threats, evaluate the effectiveness of interventions, and protect both the health and readiness of the force.

**What are the new findings?**

Mortality rates decreased from 2011 to 2014, corresponding with the drawdown of U.S. military operations in U. S. Central Command. The leading cause of death for both female and male service members was suicide and self-inflicted injury. After adjustments for age, sex, and race and ethnicity, service members had lower mortality rates than the U.S. general population, with the exception of mortality rates due to suicide and self-inflicted injury.

**What is the impact on readiness and force health protection?**

The findings of this report underscore the importance for enhancing and improving the design of suicide prevention programs within the military. While suicide is a leading cause of death for service members, the report highlights other potentially preventable causes of death that require ongoing investigation and monitoring, such as accidents and natural causes.

Military medical surveillance activities are designed and conducted to identify significant threats to the health, fitness, and operational effectiveness of military populations. Tracking mortality data, particularly the deaths of active duty service members, is crucial for maintaining force readiness. Systematic review of mortality data can detect previously unknown or under-recognized threats, inform program and policy development, guide resource allocation, and assess the effectiveness of prevention efforts.

Applicants for U.S. military service are rigorously screened to ensure physical and psychological fitness for their roles. The U.S. military provides extensive health promotion, safety, and force health protection programs to its service members in addition to offering free preventive, curative, and rehabilitative medical services. Despite these efforts, deaths from preventable injuries (e.g., combat-related, accidental, self-inflicted) remain a concern. ^1 - 4^

All-cause mortality surveillance is essential to characterize the numbers, natures, risk factors, and causes of preventable deaths among active duty service members. *MSMR* last published a comprehensive mortality report for all service branches in 2014. ^4^ From 1998 to 2011, accidental deaths were the most common manner of death for active component service members, while suicide was the most common manner of death from 2012 to 2013. ^4^ Transportation deaths declined steadily during that period, and combat-related deaths declined sharply in 2012 and 2013. ^4^ The current report provides an overview of all-cause mortality, updating previous summaries and including deaths from active as well as guard and reserve component service members of the U.S. Armed Forces from 2010 through 2020.

## Methods

The surveillance population included all individuals who served in the U.S. military during the surveillance period as a member of the active or guard and reserve components of the U.S. Army, Navy, Air Force, Marine Corps, or Coast Guard from January 1, 2010 through December 31, 2020. The outcome of interest for this report was deaths of active, guard and reserve component members while in military service. This study received a determination of ‘Not Research’ by the DHA Office of Research Protections on February 14, 2023.

Mortality data were obtained from the U.S. Department of Veterans Affairs and Department of Defense (VA / DOD) Mortality Data Repository, which compiles National Death Index (NDI) records for all veterans and military service members. These data were requested through the Defense Suicide Prevention Office (DSPO) and obtained in July 2023. That information was then merged with Defense Manpower Data Center (DMDC) demographic records, which are routinely provided for surveillance purposes to the Armed Forces Health Surveillance Division for integration within the Defense Medical Surveillance System (DMSS), to identify demographic characteristics and calculations of mortality rates. A death was considered to have occurred ‘in service’ if it occurred within the beginning and end dates (or within 90 days after last date) of a service member's DMDC demographic record. Deaths were included regardless of whether a service member was on active duty status at time of death.

Underlying causes of death were grouped into the 26 cause of death categories in the Surveillance, Epidemiology, and End Results Program (SEER) cause of death recode instructions (which include non-neoplasm causes of death). ^5^ Deaths that could not be categorized (n=1,420) by the initial 26 SEER cause of death categories were reviewed by the Armed Forces Medical Examiner System (AFMES) for additional clarification on underlying cause; if additional information was available, those deaths were then categorized as ‘other accidents and adverse effects’, ‘suicide and self-inflicted injury’, or ‘homicide and legal intervention’. Deaths determined to be due to natural or undetermined causes remained uncategorized. After review of the data, all 26 defined cause of death categories with rates less than 1 per 100,000 person-years were collapsed into a single category: ‘all other causes’. After the consolidation of cause of death categories with rates less than 1 per 100,000 person-years into the ‘all other causes’ category, 8 final cause of death categories remained: suicide and self-inflicted injury (International Classification of Diseases, 10th Revision [ICD-10]: U03, X60–X84, Y87.0), transport accidents (ICD-10: V01–V99, Y85), other accidents and adverse effects (ICD-10: W00–W99, X00–X59, Y86), neoplasm (ICD-10: C00–C97, D00–D48), operations of war (ICD-10: Y36, Y89.1), diseases of heart (ICD-10: I00–I09, I11, I13, I20–I51), homicide and legal intervention (ICD-10: U01–U02, X85–Y09, Y35, Y87.1, Y89.0), and all other causes.

Summary measures for this analysis are numbers of deaths in the surveillance population overall and mortality rates calculated as deaths per 100,000 person-years of military service. Mortality rates were summarized in relation to person-years at risk rather than individuals at risk because the U.S. military is a dynamic cohort, with many individuals entering and leaving service on any day. In any calendar year, there are many more individuals with any service than there are total person-years of active service; the latter was considered a more consistent measure of exposure to mortality risk across calendar years.

The final cause of death categories including all-cause mortality among service members were compared with mortality rates in the U.S. general population ages 15-64 years from 2010 to 2020, utilizing publicly available data sets downloaded from the U.S. Center for Disease Control and Prevention (CDC)'s National Center for Health Statistics National Vital Statistics System. ^6^ The only exception was operations of war, which was excluded from the comparison analysis since it is a uniquely military cause of death. Indirect standardization, adjusting for 5-year age category, sex, and racial or ethnic group, was used to calculate standardized mortality ratios (SMRs) and 95% confidence intervals (CIs) using a Poisson distribution. All analyses were performed using SAS ^®^ Enterprise Guide ^®^ software (version 8.3, SAS Inst., Inc., Cary, NC).

## Results

**FIGURE 1.**
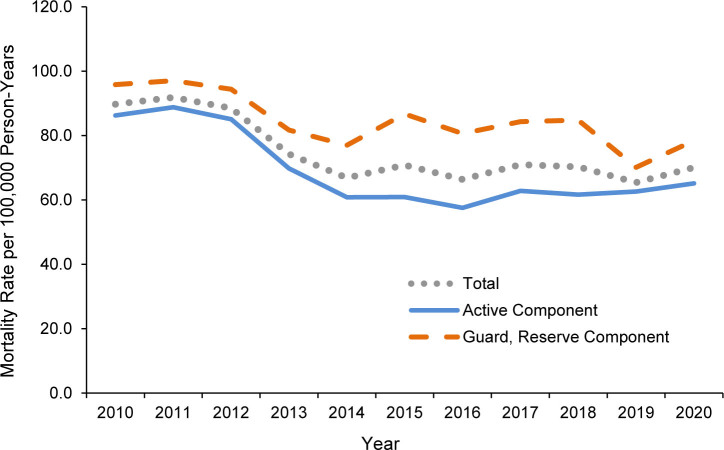
Annual All Cause Mortality Rates, Active, Guard and Reserve Components, U.S. Armed Forces, 2010–2020

From 2010 through 2020, there were 18,251 deaths among 4,956,332 U.S. military service members **(Table 1)** . The mortality rate for all components during the 11-year surveillance period was 75.3 per 100,000 person-years (p-yrs), ranging from 65.4 (in 2019) to 91.8 (in 2011). Mortality rates decreased between 2011 and 2014 for all components, although this trend was more apparent for the active component **(Figure 1)** .

**TABLE 1.** All Cause Mortality Rates ^a^ , Active, Guard and Reserve Components, U.S. Armed Forces, 2010–2020

Demographic Characteristics	Total		Active Component		Guard, Reserve Component	
	No.	Rate ^a^	No.	Rate ^a^	No.	Rate ^a^
Total	18,251	75.3	10,552	69.6	7,699	84.8
Component						
Active	10,552	69.6	—	—	—	—
Guard	4,544	91.2	—	—	—	—
Reserve	3,155	76.9	—	—	—	—
Branch of service						
Army	10,802	93.6	5,097	91.8	5,705	95.3
Navy	2,309	54.7	1,910	53.7	399	60.0
Air Force	2,863	52.3	1,678	47.3	1,185	61.5
Marine Corps	2,038	80.9	1,664	79.6	374	87.5
Coast Guard	239	48.3	203	48.2	36	49.4
Sex						
Male	16,749	83.1	9,823	76.6	6,926	94.3
Female	1,502	36.8	729	31.0	773	44.5
Race and ethnicity						
White, non-Hispanic	12,031	81.5	6,749	75.9	5,282	89.8
Black, non-Hispanic	2,804	74.8	1,616	67.8	1,188	87.0
Hispanic	1,872	57.6	1,170	54.2	702	64.3
Other	1,255	60.0	819	56.8	436	67.2
Unknown	289	74.1	198	67.6	91	93.4
Age, *y*						
<=24	6,548	77.6	4,292	74.2	2,256	85.1
25–34	6,429	69.5	3,999	66.2	2,430	75.6
35–44	3,162	66.8	1,769	64.1	1,393	70.7
45–54	1,653	102.0	441	80.7	1,212	112.8
55+	459	220.2	51	152.1	408	233.2
Sex and age, *y*						
Male, <=24	6,089	87.9	4,042	83.5	2,047	98.2
Male, 25–34	5,922	77.3	3,730	73.4	2,192	85.1
Male, 35–44	2,848	70.8	1,608	67.3	1,240	75.9
Male, 45+	1,890	121.4	443	88.2	1,447	137.2
Female, <=24	459	30.4	250	26.5	209	37.0
Female, 25–34	507	31.8	269	28.1	238	37.3
Female, 35–44	314	44.2	161	43.3	153	45.3
Female, 45+	222	81.5	49	62.9	173	89.0
Military occupation						
Combat-related	4,086	127.9	2,760	130.2	1,326	123.2
Motor transport	844	91.5	372	76.8	472	107.8
Pilot, air crew	549	70.3	391	71.5	158	67.4
Repair, engineering	4,743	71.5	2,768	62.2	1,975	90.4
Communications, intelligence	3,495	64.8	1,976	60.5	1,519	71.4
Health care	1,270	63.1	729	56.0	541	76.0
Other	3,264	61.4	1,556	51.8	1,708	73.9

Abbreviations: No., number; y, years.

a Rates per 100,000 person-years of military service.

Among all service members, compared to their respective counterparts, all-cause mortality rates were highest among the guard and reserve component (84.8 per 100,000 p-yrs), Army members (93.6 per 100,000 p-yrs), male service members (83.1 per 100,000 p-yrs), non-Hispanic White individuals (81.5 per 100,000 p-yrs), those in the oldest age category of 55 years or older (220.2 per 100,000 p-yrs), and those in combat-related occupations (127.9 per 100,000 p-yrs) **(Table 1)** ; it should be noted that serving in a combat-related occupation at time of death does not necessarily mean that a service member died while serving in combat. Among all service members, mortality rates were lowest among the active component (75.3 per 100,000 p-yrs), Coast Guard (48.3 per 100,000 p-yrs), female service members (36.8 per 100,000 p-yrs), Hispanic individuals (57.6 per 100,000 p-yrs), those aged 35-44 years (66.8 per 100,000 p-yrs), and those in other (61.4 per 100,000 p-yrs) and health care (63.1 per 100,000 p-yrs) occupations.

Suicide and self-inflicted injury was the leading cause of death during the surveillance period, accounting for 33% of all service member deaths: 25.1 per 100,000 p-yrs overall, 23.0 per 100,000 p-yrs for the active component, and 28.8 per 100,000 p-yrs for the guard and reserve component **(Table 2)** . Transport accidents and other accidents and adverse events were the second and third, respectively, leading causes of death in service members, accounting for 21% and 12% of all service member deaths, respectively **(Table 2)** . Deaths due to operations of war decreased 91% from 2010 to 2014 **(Figure 2a)** . In contrast, mortality rates due to suicide and self-inflected injury increased 28% from 2010 to 2020. Overall annual trends in mortality rates by cause of death were driven by the trends in male service members **(Figure 2b)** . Female service members had lower, more stable rates compared to male service members **(Figure 2c)** .

**TABLE 2.** Causes of Death, Active, Guard and Reserve Components, U.S. Armed Forces, 2010–2020

Category of Death	Total		Active Component		Guard, Reserve Component	
	No.	Rate ^a^	No.	Rate ^a^	No.	Rate ^a^
Suicide and self-inflicted injury	6,098	25.1	3,485	23.0	2,613	28.8
Transport accident	3,913	16.1	2,216	14.6	1,697	18.7
Other accidents and adverse effects	2,219	9.2	1,348	8.9	871	9.6
Neoplasm	1,771	7.3	776	5.1	995	11.0
Operations of war	1,265	5.2	1,154	7.6	111	1.2
Diseases of heart	1,080	4.5	476	3.1	604	6.7
Homicide and legal Intervention	738	3.0	379	2.5	359	4.0
All other causes	1,167	4.8	718	4.7	449	4.9

Abbreviation: No., number.

a Rate per 100,000 person-years of military service.

**FIGURE 2a.**
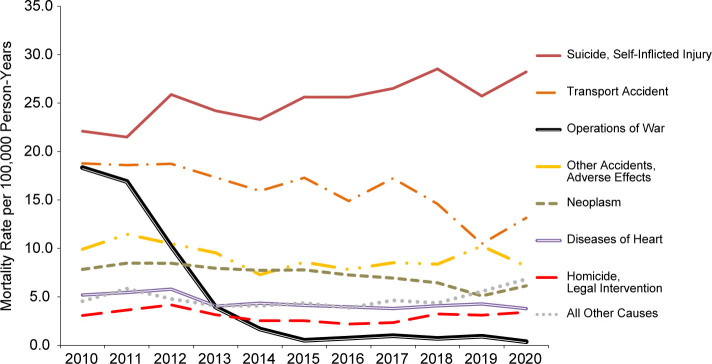
Leading Causes of Death, U.S. Armed Forces, 2010–2020

**FIGURE 2b.**
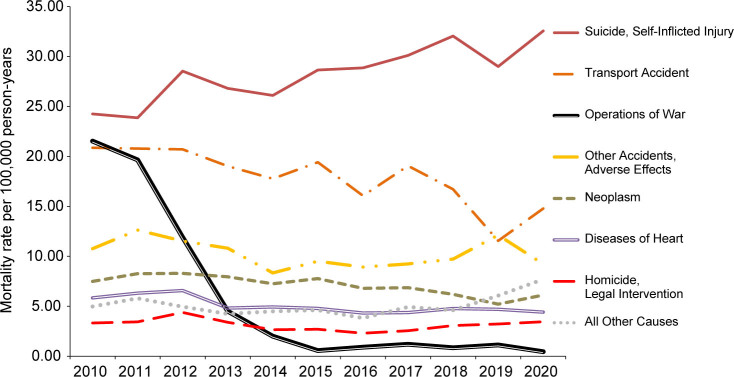
Leading Causes of Death, Male Service Members, U.S. Armed Forces, 2010–2020

**FIGURE 2c.**
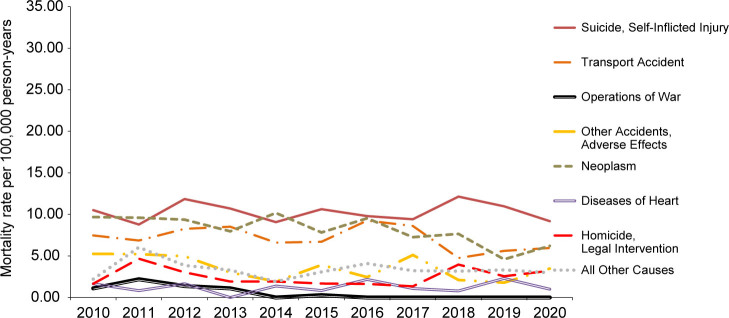
Leading Causes of Death, Female Service Members, U.S. Armed Forces, 2010–2020

The leading cause of death of male service members was suicide and self-inflicted injury (28.2 per 100,000 p-yrs), followed by transport accidents (18.0 per 100,000 p-yrs), and other accidents and adverse effects (10.3 per 100,000 p-yrs) **(Table 3)** . The leading cause of death among female service members was also suicide and self-inflicted injury (10.3 per 100,000 p-yrs), followed by neoplasms (8.1 per 100,000 p-yrs), and transport accidents (7.1 per 100,000 p-yrs) **(Table 3)** .

**TABLE 3.** Causes of Death by Sex, U.S. Armed Forces, 2010–2020

Category of Death	Men			Women		
	No.	Rate ^a^	Rank	No.	Rate ^a^	Rank
Suicide and self-inflicted injury	5,678	28.2	1	420	10.3	1
Transport accident	3,622	18.0	2	291	7.1	3
Other accidents and adverse effects	2,074	10.3	3	145	3.6	4
Neoplasm	1,439	7.1	4	332	8.1	2
Operations of war	1,243	6.2	5	22	0.5	8
Diseases of heart	1,029	5.1	6	51	1.3	7
All other causes	1,029	5.1	6	138	3.4	5
Homicide and legal intervention	635	3.2	7	103	2.5	6

Abbreviation: No., number.

a Rate per 100,000 person-years of military service.

Compared to the U. S. population, service members had significantly lower rates for all-cause mortality, transport accidents, other accidents and adverse effects, neoplasms, heart disease, and homicide **(Table 4)** . Suicide and self-inflicted injury mortality rates were higher among service members compared to the U.S. population (SMR 1.10, 95% CI 1.08, 1.13). When evaluated by sex, SMR for suicide and self-inflicted injury was 1.08 (95% CI 1.05, 1.11) for male service members and 1.66 (95% CI 1.50, 1.83) for females.

**TABLE 4.** Deaths and Mortality Rates, U.S. Military Members Compared to U.S. Population, 2010–2020

Category of Death	Observed Deaths	Expected Deaths	SMR ^a^	95% LL	95% UL	*p* -value
	No.	No.				
All-cause mortality	17,958 ^b^	42,431	0.42	0.42	0.43	<0.0001
Suicide and self-inflicted injury	6,012	5,446	1.10	1.08	1.13	<0.0001
Males	5,601	5,199	1.08	1.05	1.11	<0.0001
Females	411	247	1.66	1.50	1.83	<0.0001
Transport accident	3,851	5,074	0.76	0.74	0.78	<0.0001
Other accidents and adverse effects	2,200	8,724	0.25	0.24	0.26	<0.0001
Neoplasm	1,732	4,227	0.41	0.39	0.43	<0.0001
Diseases of heart	1,058	4,676	0.23	0.21	0.24	<0.0001
Homicide and legal intervention	729	3,597	0.20	0.19	0.22	<0.0001

Abbreviations: SMR, standardized mortality ratio; LL, lower limit; UL, upper limit.

a Adjusted for age, sex, and race and ethnicity.

b Total deaths are smaller than number shown in Table 1 because individuals with missing race information and older than age 64 years were excluded from analysis.

## Discussion

This was the first comprehensive *MSMR* mortality analysis in over a decade, and the first to include National Guard and Reserve members from all branches, including the U.S. Coast Guard. Findings from this report indicate a modestly reduced mortality rate for active component members (69.6 per 100,000 p-yrs) compared to the 1990–2011 mortality rate (75.1 per 100,000 p-yrs). ^3 - 4^ The trends in demographic risk factors for all-cause mortality were generally consistent with findings from a recent Millennium Cohort Study mortality analysis of service members and veterans deployed to post-9/11 military operations, which found that mortality rates were higher in National Guard and Reserve members, Army personnel, males, and older individuals, compared to their respective counterparts. ^7^

Overall, the leading cause of death for U.S. service members in 2020 was suicide, followed by transport accidents, and then other accidents and adverse effects. These leading 3 conditions were also the leading causes of death for service members during the combined 11-year surveillance period. Among the general U.S. population, ages 17-45 years, in 2020 the leading cause of death was unintentional injury, followed by suicide, heart disease, homicide, and malignant neoplasms. ^8^ Service member deaths due to transport accidents decreased from 2010 to 2020, whereas motor vehicle traffic death rates in the U.S. population declined between 2006 and 2010 and increased from 2010 to 2019. ^9^

According to prior *MSMR* reports, the leading causes of death in 2011 among active component service members was operations of war, followed by suicide and transport accidents. ^3 - 4^ The decline in operations of war as a leading cause of death in service members was likely influenced by drawdown of U.S. military operations in the U.S. Central Command Area of Operation during the study period. This analysis found higher rates of suicide and transport accidents in 2011, followed by operations of war, as indicated by the data presented in **Figures 2a** – **2c** . It is important to note, however, that prior *MSMR* reports only included deaths occurring while on active duty status, whereas this analysis also included deaths while not in active duty status, which likely accounts for some of the difference.

From 2010 to 2020, suicide was a leading contributor to overall deaths among U.S. service members, accounting for one-third of all deaths. The official source of suicide data in the U.S. Department of War (DOW) is the DSPO *Annual Report on Suicide in the Military* , which describes, in its most recent (2023) report, a 2013–2020 increasing gradual trend in active component suicide rates, with a decrease in 2021 followed by other increases in 2022 and 2023. ^10^ Similarly, data in the current study showed a gradual increase in suicide over the study period, with an overall 27.7% increase in suicide rates from 2011 (21.5 per 100,000 p-yrs) to 2020 (28.2 per 100,000 p-yrs) among service members in all components. The same trend was observed in the U.S. population in a report on suicide rates from 2001 to 2021, where the suicide rates from 2010 to 2020 increased during most years. ^11^ The findings of this report are also consistent with a recent U.S. Army mortality surveillance report that identified suicide as the highest cause-specific mortality rate amid a 2014–2019 decline in deaths due to natural causes. ^12^

In the 2023 DSPO report, covering a 2011-2023 surveillance period, it was reported that suicide rates among National Guard members were higher than the age- and sex-adjusted U.S. population rates, from 2012 to 2013, and suicide rates were higher among active component members compared to the age and sex adjusted U.S. population in 2020. ^10^ The current study identified similar findings in sensitivity analyses evaluating adjusted rates by sex, component, and calendar year. This study identified more suicides among service members than reported in the DSPO reports, however, particularly among National Guard and Reserve members. These differences are likely attributed to the use of VA/DOD Mortality Data Repository data to identify deaths, which is not a data source used to identify service member suicide deaths in the DSPO reports. In addition, the AFMES review of deaths that were not initially categorized in 1 of the 26 SEER cause of death categories uncovered more suicide deaths than would have been identified in this report, based on VA/DOD Mortality Data Repository data alone. This emphasizes the importance of comprehensive and integrated death data review.

During the surveillance period, male service members' suicide mortality rate was more than double that of their female counterparts, a finding that contrasts sharply with the civilian population, where men's suicide rate is 4 times greater than women's. ^13^ In fact, when compared to an age- and race-adjusted U.S. population, this study found that female service members had a 66% increased rate of suicide, whereas male service members had only an 8% increased rate. This finding does not, however, establish a causal link between military service and suicide risk, as suicide causes could not be determined from available data. This finding does, however, identify women as a specific subpopulation for the DOW to refine and more effectively target future suicide prevention efforts and resources. At least 1 prior study identified higher suicide incidence among active component women ages 17-29 years compared to age-adjusted rates in the U.S. population in 2010, 2012, and 2014. ^14^ This trend has also been observed in veterans: In the *2025 National Veteran Suicide Prevention Annual Report* the suicide rate in 2023 for female veterans was 103.1% higher than for non-veteran female U.S. adults, while the age-adjusted rate for male veterans was 49.7% higher than for non-veteran male U.S. adults. ^15^

Service members tend to be healthier than the general U.S. population, due to a number of factors including medical screening prior to service accession (disqualifying individuals with significant medical conditions), multiple programs to maintain the health of the force (e.g., physical fitness standards, safety and force health protection programs, no-cost medical services), and frequent attrition (e.g., medical disability) of service members who develop life-threatening medical conditions prior to terminal illness stages. ^16^ Consequently, it was not surprising that, with the exception of suicide, death rates for conditions evaluated in this study (e.g., neoplasms, heart diseases) were lower for service members than those in the U.S. population, even after adjustments for age, sex, and race and ethnicity. Similarly, although guard and reserve component members are generally required to meet the same physical fitness standards as active duty members, they may test less frequently and face different daily training accountability. Guard and reserve component members may also have less consistent health insurance coverage and less on-base support, which could contribute to the higher mortality rates observed in guard and reserve component members in this analysis.

Limitations of this mortality review include use of NDI records contained in the VA/DOD Mortality Data Repository as the primary source of data for cause and manner of death, with the exception of the uncategorized records (n=1,420) reviewed by AFMES. Previous *MSMR* reports utilized data maintained by AFMES, which historically collected source documents from civilian jurisdictions on all active duty deaths, which were then validated, coded, and analyzed. ^4^ AFMES has since lost that capability; efforts are underway to restore it. The use of a different source of mortality data in this report makes it more challenging to compare these results to the findings of prior reports.

This analysis is intended to provide a survey of recent trends in service member mortality rates, without details of types of intentional or unintentional deaths. It should be noted, however, that use of firearms has consistently been described as the most common method of death in service members who died by suicide, according to annual DSPO reports. ^10^ Also of note, the Fiscal Year 2024 National Defense Authorization Act (NDAA) (Public Law 118-31) requires an annual report on fatal and non-fatal drug overdoses by members of the U.S. Armed Forces. ^17^ The most recent NDAA report, released in April 2025, indicates that both fatal and non-fatal overdoses were lower in service members compared to the U.S. population, likely due to frequent, random urinalysis drug testing combined with anti-drug education and outreach. Furthermore, the NDAA report reveals that fatal and non-fatal drug overdoses in service members decreased by more than 40% from 2021 to 2023. ^17^

The current study was limited to deaths that occurred while in military service; mortality rates for veterans and former service members were not assessed. Deaths were included if they occurred within 90 days after a service member's last service record, to account for imprecision in military separation dates. Some deaths that occurred shortly after a service member separated from service may have been inadvertently included. This study also did not evaluate multiple or secondary causes of death. Results are dependent upon the accuracy of the information record of the underlying cause of death, which can be subjective. When comparing mortality rates with the U.S. population, U.S. population data used the age category of 15-19 years, which is not the same as the military service member age category of 17-19 years, and may have resulted in some residual confounding. In addition, service members could not be excluded from the U.S. population comparison group.

By nature, many military activities are dangerous and sometimes life-threatening. Mortality surveillance is a key component of comprehensive health surveillance among a military population. The findings of this report underscore the importance of suicide prevention programs within the military, with the consideration of the unique risk factors affecting women in service. Further research would be required to better understand why certain subgroups, such as Army soldiers and National Guard members, have higher mortality rates. Continued, detailed mortality surveillance remains essential to identifying emerging threats, evaluating the effectiveness of interventions, and ultimately protecting the health and readiness of the force.
